# Different modulation of short‐ and long‐latency interhemispheric inhibition from active to resting primary motor cortex during a fine‐motor manipulation task

**DOI:** 10.14814/phy2.12170

**Published:** 2014-10-07

**Authors:** Takuya Morishita, Shinji Kubota, Masato Hirano, Kozo Funase

**Affiliations:** 1 Human Motor Control Laboratory, Graduate School of Integrated Arts and Sciences, Hiroshima University, Higashi‐Hiroshima, Japan

**Keywords:** Corpus callosum, fine‐motor task, interhemispheric inhibition, transcranial magnetic stimulation

## Abstract

Performing a complex unimanual motor task markedly increases activation not only in the hemisphere contralateral to the task‐performing hand but also in the ipsilateral hemisphere. Transcranial magnetic stimulation studies showed increased motor evoked potential amplitude recorded in resting hand muscles contralateral to the task‐performing hand during a unimanual motor task, and transcallosal inputs from the active hemisphere have been suggested to have responsibilities for this phenomenon. In the present study, we used a well‐established double‐pulse transcranial magnetic stimulation paradigm to measure two phases of interhemispheric inhibition from the active to the resting primary motor cortex during the performance of a complex unimanual motor task. Two different unimanual motor tasks were carried out: a fine‐motor manipulation task (using chopsticks to pick up, transport, and release glass balls) as a complex task and a pseudo fine‐motor manipulation task as a control task (mimicking the fine‐motor manipulation task without using chopsticks and picking glass balls). We found increased short‐latency interhemispheric inhibition and decreased long‐latency interhemispheric inhibition from the active to the resting primary motor cortex during the fine‐motor manipulation task. To the best of our knowledge, the present study is the first to demonstrate different modulation of two phases of interhemispheric inhibition from the active to the resting primary motor cortex during the performance of a complex unimanual motor task. The different modulation of short‐ and long‐latency interhemispheric inhibition may suggest that two phases of interhemispheric inhibition are implemented in distinct circuits with different functional meaning.

## Abstract (precis)

Different modulation of two phases of IHI was obtained during the complex unimanual task. The timing of TMS trigger did not affect the MEP amplitude and the degree of IHI. The results of the present study suggest that SIHI and LIHI may have different functional meaning.

## Introduction

Performing a unimanual motor task leads to activation not only in the hemisphere contralateral to the task‐performing hand but also in the ipsilateral hemisphere. Neuroimaging studies demonstrated activation of the ipsilateral hemisphere especially the primary motor cortex (M1) area during a unimanual motor task (Kim et al. 1993; Hummel et al. 2003; Verstynen et al. 2005). Similarly, transcranial magnetic stimulation (TMS) studies showed increased motor evoked potential (MEP) amplitude recorded in resting hand muscles contralateral to the task‐performing hand (i.e., TMS was delivered to the M1 ipsilateral to the task‐performing hand) during a unimanual motor task (Stedman et al. 1998; Muellbacher et al. 2000; Stinear et al. 2001). The ipsilateral activity has been thought to be mediated through the corpus callosum (Kobayashi et al. 2003) and in accordance with this view, several studies have investigated interhemispheric interactions during the performance of a unimanual motor task – particularly between two M1 regions in TMS studies.

A double‐pulse TMS paradigm enables us to investigate the neural mechanisms of the corpus callosum, which is involved in interhemispheric inhibition (IHI). The principle of the double‐pulse TMS paradigm for measuring IHI is to see the effects of applying a conditioning stimulus (CS) to one hemisphere on the size of MEP amplitude evoked by the application of a test stimulus (TS) to the opposite hemisphere (Ferbert et al. 1992). Two phases of IHI have been reported: short‐latency IHI (SIHI) at interstimulus intervals (ISI) between the CS and the TS of ~10 msec and long‐latency IHI (LIHI) at ISIs of 40–50 msec (Chen et al. 2003; Ni et al. 2009). Several studies have provided evidence that SIHI is predominantly mediated through the corpus callosum (Wahl et al. 2007; Li et al. 2013). The role of IHI is still inconclusive; one theory is that the role of SIHI is to suppress involuntary activity (Hübers et al. 2008). However, the mechanisms of LIHI and its role are still unknown. One of the reasons is that LIHI has not been examined in detail especially during the performance of a complex motor task.

Based on findings of previous studies, SIHI and LIHI have shown different modulation under various conditions. For example, a pharmacological study demonstrated that LIHI was mediated by GABA_B_ receptors (Irlbacher et al. 2007), but the exact neurotransmission of SIHI is still inconclusive. In addition, different modulation of SIHI and LIHI was found in patients with neurophysiological conditions such as Parkinson's disease and multiple sclerosis (Li et al. 2007; Wahl et al. 2011). However, modulation of LIHI during a unimanual motor task was unclear (Talelli et al. 2008a; Uehara et al. 2014). In our previous study, we reported increased SIHI from the active to the resting M1 during a complex unimanual motor task (Morishita et al. 2012). Increased ipsilateral M1 excitability was evident during the complex unimanual task compared with a simple unimanual task, therefore, we concluded that the increased SIHI while performing the complex task was also due to complexity of the task – task‐dependent modulation. We hypothesized that clear modulation of LIHI might also be obtained during the performance of a complex unimanual task; we also expected different modulation of SIHI and LIHI based on the previous studies. To explore these hypotheses, we adopted the same task from our previous study and examined the effects of performing a complex unimanual motor task on two phases of IHI between M1 regions and compared them with those induced during a simple unimanual motor task.

## Experimental Procedure

### Subjects

A total of 13 right‐handed subjects (four female; mean age 24.2 ± 2.7 years, age range 21–29 years) gave their informed written consent to participate in this study. The handedness of each subject was confirmed with the Edinburgh Handedness Inventory (lateralization index >0.7) (Oldfield 1971). The subjects were comfortably seated on a chair and were instructed to put both of their hands on a horizontal plate attached to the chair's armrests. All experimental procedures were carried out in accordance with the Declaration of Helsinki and were approved by the ethics committee of Hiroshima University.

### EMG recording

Surface electromyography (EMG) recordings were taken from the first dorsal interosseous (FDI) muscles of both hands with Ag/AgCl surface electrodes with a diameter of 9 mm. EMG recordings were amplified at a bandwidth of 5 Hz–3 kHz, and all amplification procedures were controlled with a signal processor (model 7S12, NEC San‐ei Co. Ltd, Tokyo, Japan). The analog outputs from the signal processor were digitized at a sampling rate of 10 kHz and then transferred to a computer for offline analysis (PowerLab system, Scope version 3.7.6, AD Instruments Pty. Ltd, Sydney, Australia). Supramaximal electrical stimuli, 1 msec square pulses, were delivered via paired bar‐type electrodes at 1 sec intervals to the ulnar nerve in the left wrist to obtain maximum muscle responses (Mmax). Recordings of the integrated EMG of the FDI in the task‐performing hand for the 100 msec prior to the TMS trigger were made with the Integral Abs, Scope (version 3.7.6), PowerLab system.

### TMS and assessment of IHI

Two figure‐of‐eight coils (70 mm in diameter) were separately connected to two MagStim200 stimulators (Magstim). The TS for measuring IHI was applied to the left M1 and the CS was applied to the right M1. The optimal position over the left M1 for evoking MEP from the right FDI was determined with the handle pointing backward and rotated ~45° away from the mid‐sagittal line and the induced current in the brain was in the anterior‐medial direction. The CS coil over the right M1 was placed perpendicular to the mid‐sagittal line to prevent two coils from overlapping. The optimal position over the right M1 for evoking MEP from the left FDI was determined with this coil orientation and it induced medially directed current in the brain. The optimal positions were marked on a nylon mesh swimming cap worn by the subjects with a soft‐tip pen to ensure reliable coil placement between trials. The optimal position was defined as the location where a magnetic pulse evoked the largest MEP amplitude from the contralateral FDI muscle. The resting motor threshold (RMT) of the contralateral FDI was defined as the minimum TMS intensity required to evoke MEP of >50 *μ*V in at least 5 of 10 trials in each M1. If it was not possible to place both coils with the abovementioned setup, the CS coil was placed tangentially to the scalp with the handle pointing forward and rotated ~15° away from the mid‐sagittal line. In this case, the CS intensity was still calculated with the original coil orientation. We used this setup because Chen et al. (2003) and Ni et al. (2009) reported that the current direction of the CS did not affect the degree of IHI.

### Hand motor tasks

All subjects performed two different unimanual motor tasks. A fine‐motor manipulation (FM) task (Morishita et al. 2011, 2012) was conducted in order to investigate the neural mechanisms involved in performing a complex unimanual motor task. The subjects were instructed to pick up a glass ball (diameter 15 mm, weight 5.6 g) with custom‐made wooden chopsticks (length 240 mm, weight 4.6 g, dimensions of the rectangular tip: 5 × 3 mm) from one box (150 × 220 × 60 mm) that was placed on a table. They then had to transport and release it into another box that was also placed on the table. They were instructed not only to repeat this series of movements for ~3 min as accurately and quickly as possible but also at a comfortable pace. The two boxes were placed on a horizontal plate attached to the chair's armrests. In addition, in order to compare the effects of properties of the task with those of the FM task, the subjects also performed a pseudo‐FM (pFM) task (Morishita et al. 2011). The pFM task involved a repetitive grasping movement of fingertip using their index finger, middle finger, and thumb as a comparative simple task without manipulation of the chopsticks. The subjects continuously repeated each grasping movement phase (i.e., grasping and releasing) according to a beeping sound delivered at 1 Hz. They were also instructed to produce similar EMG activity to that measured in the FM task. Before TMS measurements began, the subjects practiced both tasks for ~1 min. Special attention was paid to involuntary EMG activity of the resting right FDI during the performance of the hand motor tasks. EMG activity of the resting right FDI was monitored on a computer screen and auditory feedback was given to the subjects via a loudspeaker to provide the state of muscle relaxation. Both tasks were performed with the nondominant left hand because our previous study showed performing the FM task with the nondominant left hand demonstrated larger increased MEP amplitude recorded in the contralateral FDI muscle compared with that obtained when the task‐performing hand was the dominant right hand (Morishita et al. 2011).

During the hand motor tasks in all experiments except Experiment 4, TMS was manually triggered, regardless of the movement phase of the tasks, with careful attention paid to ensure that magnetic pulses were not delivered during a certain movement phase. We mainly used this procedure because we confirmed that the changes in MEP amplitude in the resting FDI detected during the FM task were not related to the timing of TMS trigger in our previous studies (Morishita et al. 2011, 2012). The subjects were instructed to ignore stimulus‐induced responses as much as possible.

### Experiment 1: Effects of performing hand motor tasks on MEP amplitude in contralateral resting FDI

**Figure 1.**
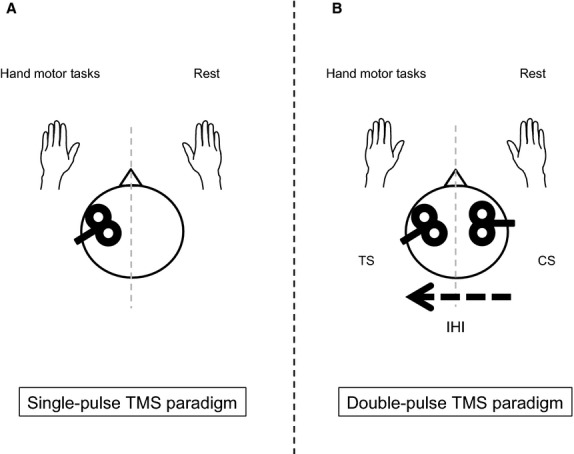
Schematic illustration of experimental paradigms in the present study. Hand motor tasks were carried out with the left hand. (A) Single‐pulse transcranial magnetic stimulation (TMS) paradigm. TMS was delivered to the left primary motor cortex (M1) and motor evoked potential (MEP) was recorded in the right first dorsal interosseous (FDI). (B) Double‐pulse TMS paradigm. The conditioning stimulus (CS) for measuring interhemispheric inhibition (IHI) was delivered to the right M1 and the test stimulus (TS) was delivered to the left M1. Conditioned MEP amplitude recorded in the right FDI was considered as the degree of IHI. During the hand motor tasks, IHI from the active (right M1) to the resting M1 (left M1) was measured.

All subjects participated in Experiment 1. In Experiment 1, we examined the effects of performing the FM task and the pFM task on MEP amplitude recorded in the resting FDI contralateral to the task‐performing hand, using the single‐pulse TMS paradigm. Figure 1 shows schematic illustration of experimental paradigms in the present study. TMS was delivered to the left M1 ipsilateral to the task‐performing hand (Fig. 1A). We carried out the experiment in the following three conditions: resting conditions in both hands (REST), performing the FM task with the left hand (FM task), and performing the pFM task with the left hand (pFM task). The stimulus intensity was set to evoke MEP ~1 mV when both hands were completely relaxed. In Experiment 1, the FM task was always performed prior to the pFM task so that the muscle activity observed during the pFM task could be ensured producing similar integrated EMG values to those induced during the FM task. Fifteen to 18 TMS trials were performed in each condition. The stimuli were delivered at intervals of 5–8 sec.

### Experiment 2: Changes in SIHI and LIHI during hand motor tasks

Ten subjects (four female; mean age 24.7 ± 2.8 years, range 22–29 years) participated in Experiment 2. In Experiment 2, we examined changes in two phases of IHI from the active to the resting M1 during the FM task and the pFM task, using the double‐pulse TMS paradigm. The TS for measuring IHI was delivered to the left M1 and the CS was delivered to the right M1 (Fig. 1B). We adopted two ISIs between the CS and the TS: 10 msec for SIHI and 50 msec for LIHI (Ferbert et al. 1992; Gerloff et al. 1998; Ni et al. 2009). The three conditions – REST, FM task, and pFM task – for Experiment 2 and the test order of SIHI and LIHI were randomized between subjects, although REST was always performed prior to the hand motor tasks in order to determine the CS intensity used in Experiment 2. The CS intensity was adjusted to produce 30–40% inhibition of the MEP evoked by the TS alone in REST for SIHI and LIHI, respectively. The CS intensity was initially set to 110–120% of RMT of the MEP in the left FDI and adjusted if necessary. The TS intensity was adjusted to evoke a control MEP of 1–1.5 mV in each condition. Each condition consisted of TS alone trials and double‐pulse TMS trials. Fifteen to 18 TMS trials were performed for the TS alone and double‐pulse TMS trials, respectively. SIHI and LIHI during the hand motor tasks were tested on the same day except for those who participated in Experiment 4 (please see below). The stimuli were delivered at intervals of 5–8 sec.

### Experiment 3: Effects of CS intensity on degree of SIHI and LIHI during hand motor tasks

Eight subjects (two female; mean age 23.9 ± 2.0 years, range 21–27 years) participated in Experiment 3. Five subjects also participated in Experiment 2. In Experiment 2, we adjusted the CS intensity to an individual level and different modulation of SIHI and LIHI was obtained (please see Results). In order to rule out the CS intensity as a potential factor for the results in Experiment 2, we examined the effects of different CS intensities on the degree of SIHI and LIHI during the hand motor tasks. In Experiment 3, three CS intensities, 1.0, 1.2, and 1.4 times the RMT of the MEP in the left FDI, were tested. Each condition consisted of TS alone trials and double‐pulse TMS trials with three randomly selected CS intensities. Fifteen to 20 TS alone trials were performed for the TS alone trials and 12–15 double‐pulse TMS trials were performed for each CS intensity. The three conditions – REST, FM task, and pFM task – for Experiment 3, the test order of SIHI and LIHI, and the test order of different CS intensities were randomized between subjects. Other experimental procedures were same as Experiment 2 mentioned earlier.

### Experiment 4: Effects of timing of TMS on SIHI and LIHI during FM task

Six subjects (two female; mean age 25.2 ± 2.6 years, range 22–29 years) who participated in Experiment 2 also participated in Experiment 4. In Experiment 4, we examined the effects of timing of TMS trigger on MEP amplitude in the resting right FDI and the degree of SIHI and LIHI during the FM task. In our previous study, we demonstrated that timing of TMS trigger did not affect the degree of SIHI (Morishita et al. 2012). However, we could not exclude the possibility that timing of TMS trigger might affect the degree of LIHI. Based on findings of previous studies, SIHI and LIHI have shown different modulation under various conditions. Therefore, we also examined the effects of timing of TMS trigger on the excitability of transcallosal inputs in order to see differences between SIHI and LIHI if they existed. We used a similar method that was used in our previous study (Morishita et al. 2012). During the performance of the FM task, TMS was triggered automatically in response to the force signal from a foil strain gauge (type N11‐FA‐5‐120‐11‐VSE3; NEC San‐ei Instruments) that was attached to the custom‐made chopsticks. The force signal was amplified by a strain amplifier (model 6M92; NEC San‐ei) that was connected to the strain gauge. A force level for triggering TMS was set individually (~0.3 N) so that TMS would be triggered when the glass ball was pinched. The CS was delivered to the right M1 at one of four intervals (0, 200, 400, and 600 msec) after the force signal had reached the triggering level. The TS was then delivered to the left M1 after the CS at ISIs of 10 msec for SIHI and 50 msec for LIHI. A triggered point at 0 msec corresponded to a pinching phase of the task (i.e., right after the subjects pinched a glass ball with the chopsticks) and at 600 msec almost corresponded to a release‐preparation phase in most subjects (i.e., after the subjects released the glass ball). Triggered points at 200 and 400 msec corresponded to transporting phases of the task. The TS intensity was adjusted to evoke a control MEP of 1–2 mV when the triggered timing was at 200 msec. We initially set the triggered timing at 200 msec and the TS intensity that was used in Experiment 2. The TS intensity was adjusted if evoked MEP amplitude was not 1–2 mV after ~7 MEPs were evoked. The CS intensity that was used in Experiment 4 was same as in Experiment 2. SIHI and LIHI were tested on separate days and the test order of SIHI and LIHI were randomized between subjects. Fifteen to 20 TS alone trials and double‐pulse TMS trials were performed for each of the four intervals. The stimuli were delivered at intervals of at least 6 sec.

### Statistical analysis

The peak‐to‐peak MEP amplitude for each trial was measured offline. In Experiment 1, one‐way ANOVA with repeated measures was performed to analyze the mean MEP amplitude and the mean integrated EMG value recorded in the resting FDI among the task conditions (REST, FM task, and pFM task). The integrated EMG value recorded in the task‐performing left FDI was expressed as a percentage of the maximum voluntary contraction value and analyzed between FM task and pFM task with the paired *t*‐test. For the double‐pulse TMS paradigm, the MEP amplitude recorded in the resting right FDI was expressed as a percentage of the mean test MEP amplitude evoked by the TS alone ([CS + TS]/TS alone × 100) for all analyses. Values below 100 indicate inhibition and values above 100 indicate facilitation. In Experiments 2 and 3, two‐way ANOVA with repeated measures was used to compare the test MEP amplitude evoked by the TS alone (ISI × TASK). In Experiment 2, two‐way ANOVA with repeated measures was performed to analyze the degree of IHI (ISI × TASK). In Experiment 3, three‐way ANOVA with repeated measures was performed to analyze IHI (ISI × TASK × CS INTENSITY). If a significant main effect was found, post hoc tests for multiple comparisons Fisher's protected least significant difference (PLSD) test were performed among the task conditions. In Experiment 4, two‐way ANOVA with repeated measures was performed to analyze the test MEP amplitude, the degree of IHI, and the amplitude of MEP evoked by the CS (CS MEP) (ISI × TRIGGER TIMING) to reveal the effects of timing of TMS trigger. The CS MEP was expressed as a percentage of the Mmax (CS MEP/Mmax × 100). Greenhouse–Geisser correction was used and corrected *P* values were reported in case of nonsphericity. The threshold for significance was set at *P* < 0.05. If EMG activity of >25 *μ*V exceeding from the baseline was detected in the resting FDI, the data were omitted from the analyses. As a result, 93.5% of trials were used for the subsequent analyses (Experiment 1: 95.4%; Experiment 2: 93.4%; Experiment 3: 94.6%; Experiment 4: 91.2%). All data are expressed as mean ± standard deviation (SD).

## Results

### Experiment 1: Effects of performing hand motor tasks on MEP amplitude in contralateral resting FDI

**Figure 2.**
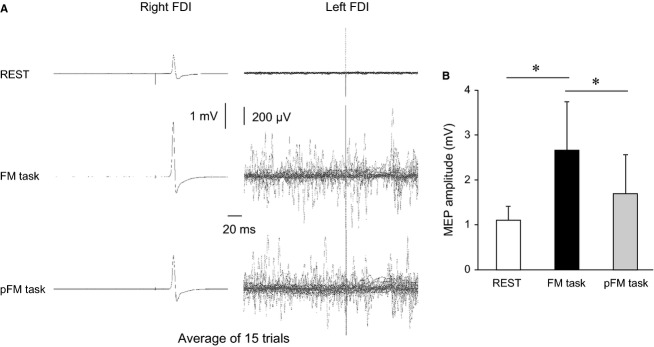
(A) Typical averaged MEP waveforms (*n* = 15) recorded in the resting right FDI and superimposed electromyography (EMG) recordings (*n* = 15) recorded in the active left FDI in one subject in each condition (REST, fine‐motor manipulation [FM] task, and pseudo‐FM [pFM] task). (B) The mean MEP amplitude values (*n* = 13, ±standard deviation: SD) recorded in the resting right FDI in each condition (REST, FM task, and pFM task). The *y*‐axis indicates the MEP amplitude. The open, closed, and gray bars indicate REST, FM task, and pFM task, respectively. The asterisks (*) indicate significant differences (*P* < 0.01).

The RMT for the left M1 and the right M1 for all subjects were 45.4 ± 6.7% and 50.6 ± 10.9% of maximum stimulator output. Figure 2A shows the typical averaged MEP waveforms (*n* = 15) recorded in the resting right FDI and superimposed EMG (*n* = 15) in the active left FDI in one subject at rest and during the hand motor tasks with the left hand. The mean MEP amplitude values obtained in these conditions are shown in Figure 2B. A significant main effect of the task conditions was detected (*F*_2,24_ =12.956, *P* < 0.001). Post hoc comparisons revealed significant differences between REST and FM task and between FM task and pFM task (*P* < 0.01). The mean integrated EMG values of the FDI in the resting hand were 0.457 ± 0.18 mV.msec at rest, 0.552 ± 0.20 mV.msec during the FM task, and 0.526 ± 0.22 mV.msec during the pFM task. There were no significant differences among the conditions (*F*_2,24_ = 0.777, *P* = 0.468). The mean integrated EMG values of the FDI in the task‐performing hand were 5.54 ± 2.48% of maximum voluntary contraction during the FM task and 5.17 ± 4.26% of maximum voluntary contraction during the pFM task. There was no significant difference in integrated EMG values between the hand motor tasks (*t* = 0.342, df = 12, *P* = 0.739).

### Experiment 2: Changes in SIHI and LIHI during hand motor tasks

**Figure 3.**
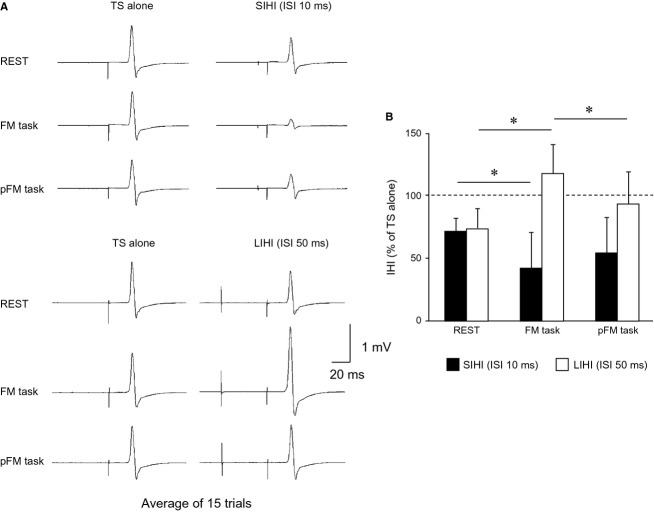
(A) Typical averaged MEP waveforms (*n* = 15) recorded in the resting right FDI showing the MEP amplitude evoked by the TS alone (left) and the degree of IHI (right) in one subject in each condition (REST, FM task, and pFM task). (B) The mean IHI values (*n* = 10, ±SD) recorded in the resting right FDI in each condition (REST, FM task, and pFM task). The *y*‐axis indicates the amplitude of the conditioned MEP amplitude expressed as a percentage of the mean test MEP amplitude evoked by the TS alone showing the degree of IHI. The dashed line indicates the MEP amplitude evoked by the TS alone (100%). Values below 100% indicate inhibition and values above 100% indicate facilitation. The closed and open bars indicate interstimulus intervals (ISIs) of 10 msec (short‐latency IHI: SIHI) and 50 msec (long‐latency IHI: LIHI), respectively. The asterisks (*) indicate significant differences (*P* < 0.05).

The CS intensities for producing 30–40% inhibition of the MEP evoked by the TS alone were 114.5 ± 10.9% of RMT for SIHI and 114.5 ± 13.6% of RMT for LIHI. Figure 3A shows the typical averaged waveforms (*n* = 15) recorded in the resting right FDI showing the MEP amplitude evoked by the TS alone (left) and the degree of IHI (right). The mean TS intensities and the mean MEP amplitude evoked by the TS alone in Experiment 2 were summarized in Table 1. There were no significant effects of ISI and TASK on the MEP amplitude evoked by the TS alone (ISI: *F*_1,9_ = 0.260, *P* = 0.617; TASK: *F*_2,18_ = 0.144, *P* = 0.866), indicating that the MEP amplitude evoked by the TS alone was well adjusted. There was also no significant ISI × TASK interaction (*F*_2,18_ = 0.468, *P* = 0.630). Figure 3B shows the mean IHI values recorded in the resting right FDI at rest and during the hand motor tasks. Under the well‐controlled MEP amplitude evoked by the TS alone, two‐way ANOVA revealed a significant effect of ISI (*F*_1,9_ = 22.118, *P* < 0.001) but no significant effect of TASK (*F*_2,18_ = 1.145, *P* = 0.330) on IHI. There was also a significant ISI × TASK interaction (*F*_2,18_ = 24.470, *P* < 0.001), indicating that the significant effects of ISI were different depending on the task conditions. Separate one‐way ANOVA showed that significant effects of TASK for both ISIs (*F*_2,18_ = 3.714, *P* = 0.038 for SIHI; *F*_2,18_ = 10.374, *P* < 0.001 for LIHI). In the post hoc tests, significant differences between REST and FM task were detected for both ISIs (*P* < 0.05). There was also a significant difference between FM task and pFM task for LIHI (*P* < 0.05).

**Table 1.** Summary of test MEP amplitude and TS intensity (% of RMT) in each condition.

	SIHI (ISI 10 msec)			LIHI (ISI 50 msec)		
	REST	FM task	pFM task	REST	FM task	pFM task
Experiment 2 (*n* = 10)	1.16 ± 0.31 (128.0 ± 11.4)	1.17 ± 0.27 (112.4 ± 7.1)	1.22 ± 0.35 (119.5 ± 7.3)	1.31 ± 0.46 (124.8 ± 12.7)	1.21 ± 0.30 (111.9 ± 7.0)	1.21 ± 0.33 (117.7 ± 6.2)
Experiment 3 (*n* = 8)	1.27 ± 0.39 (128.2 ± 11.6)	1.42 ± 0.31 (110.6 ± 6.6)	1.22 ± 0.37 (122.9 ± 7.2)	1.35 ± 0.33 (126.5 ± 11.5)	1.23 ± 0.21 (110.3 ± 6.9)	1.21 ± 0.29 (120.7 ± 7.1)

ISI, interstimulus interval; LIHI, long‐latency interhemispheric inhibition; SIHI, short‐latency interhemispheric inhibition; TS, test stimulus. Values (in mV, with % of resting motor threshold [RMT] in parentheses) are mean ± SD.

### Experiment 3: Effects of CS intensity on degree of SIHI and LIHI during hand motor tasks

**Figure 4.**
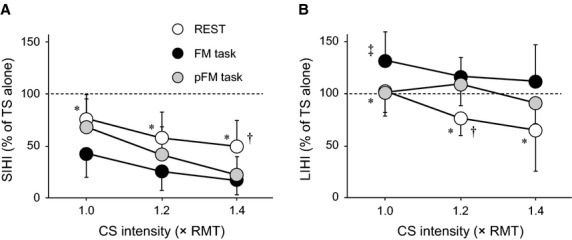
(A, B) The mean IHI values (*n* = 8, ±SD) recorded in the resting right FDI in each condition (REST, FM task, and pFM task). The open, closed, and gray circles indicate REST, FM task, and pFM task, respectively. Two ISIs were tested: 10 msec for SIHI (A) and 50 msec for LIHI (B). The *y*‐axis indicates the conditioned MEP amplitude expressed as a percentage of the mean test MEP amplitude evoked by the TS alone showing the degree of IHI. The *x*‐axis indicates the CS intensity (ratio to the resting motor threshold: RMT). The dashed line indicates the MEP amplitude evoked by the TS alone (100%). Values below 100% indicate inhibition and values above 100% indicate facilitation. The asterisks (*) indicate significant differences (*P* < 0.05) between REST and FM task. The daggers (†) indicate significant differences (*P* < 0.05) between REST and pFM task. The double dagger (‡) indicates a difference (*P* < 0.05) between FM task and pFM task.

The mean TS intensities and the mean MEP amplitude evoked by the TS alone in Experiment 3 are summarized in Table 1. Similar to Experiment 2, two‐way ANOVA for the MEP amplitude evoked by the TS alone revealed no significant effects of ISI and TASK (ISI: *F*_1,7_ = 0.113, *P* = 0.742; TASK: *F*_2,14_ = 0.782, *P* = 0.467). Additionally, there was no significant ISI × TASK interaction (*F*_2,14_ = 0.969, *P* = 0.392). Results of three‐way ANOVA are summarized in Table 2. Importantly, there was a significant ISI × TASK interaction, indicating that the significant effects of ISI were different depending on the task conditions. Figure 4 shows the mean IHI values for each ISI (A: 10 msec for SIHI; B: 50 msec for LIHI) recorded in the resting right FDI at rest and during the hand motor tasks. Separate two‐way ANOVA showed significant effects of TASK and CS INTENSITY for both ISIs (TASK: *F*_2,14_ = 6.088, *P* = 0.008 for SIHI; *F*_2,14_ = 11.018, *P* < 0.001 for LIHI) (CS INTENSITY: *F*_2,14_ = 29.473, *P* < 0.001 for SIHI; *F*_2,14_ = 5.254, *P* =0.009 for LIHI). Additionally, there were no significant TASK × CS INTENSITY interactions for both ISIs (*F*_4,28_ = 1.127, *P* = 0.357 for SIHI; *F*_4,28_ = 1.259, *P* =0.301 for LIHI). Post hoc comparisons showed differences among the task conditions (Fig. 4), which is similar to the results of Experiment 2. The results of Experiments 2 and 3 indicate that different modulation of SIHI and LIHI was obtained during the FM task: increased SIHI and decreased LIHI.

**Table 2.** Results of three‐way repeated measures ANOVA with factors of “ISI,” “TASK,” and “CS INTENSITY” on IHI.

	df	*F* value	*P* value
ISI	1	40.124	**<0.001**
TASK	2	0.257	0.777
CS INTENSITY	2	37.972	**<0.001**
ISI × TASK	2	27.413	**<0.001**
ISI × CS INTENSITY	2	2.240	0.171
TASK × CS INTENSITY	4	0.524	0.719
ISI × TASK × CS INTENSITY	4	1.548	0.216

CS, conditioning stimulus. Significant values are given in bold.

### Experiment 4: Effects of timing of TMS on SIHI and LIHI during FM task

**Figure 5.**
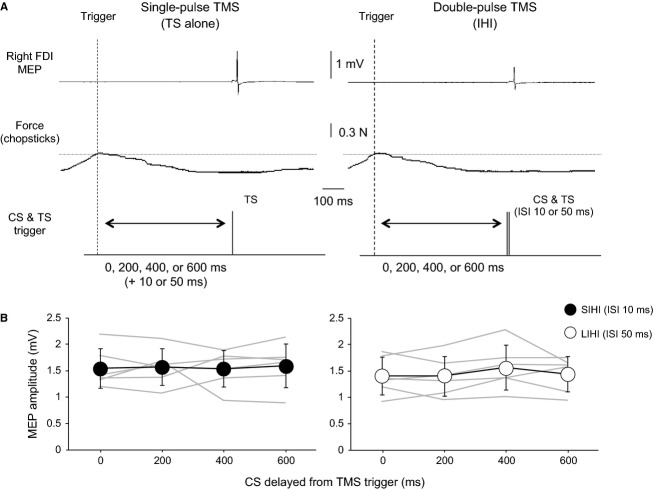
(A) Example of single‐pulse TMS (left) and double‐pulse TMS (right) during the FM task: typical averaged MEP waveforms (*n* = 15) recorded in the resting right FDI. Left: MEP amplitude evoked by the TS alone. Right: MEP amplitude recorded after the application of both the CS and the TS showing the degree of IHI. Averaged force curves (*n* = 15) measured by a foil strain gauge attached to a custom chopstick during the FM task are shown. Horizontal dotted lines indicate the triggering level (~0.3 N). The CS and the TS were triggered when the force reached the triggering level. Vertical dashed lines indicate the timing of TMS trigger. Four intervals between the triggering level being reached and the initiation of the TMS trigger timing were tested (0, 200, 400, and 600 msec). Typical waveforms and force curves are shown when the timing of TMS trigger is at 600 msec. (B) Time courses of the mean MEP amplitude values (*n* = 6, ±SD) observed in the different timing of TMS trigger for SIHI and LIHI, respectively. Individual data from six subjects are shown in gray lines. The *y*‐axis indicates the MEP amplitude recorded in the resting right FDI and the *x*‐axis indicates the different timing of TMS trigger.

Figure 5A shows examples of the single‐pulse TMS paradigm (Fig. 5A, left) and the double‐pulse TMS paradigm (Fig. 5A, right) in Experiment 4. TMS was automatically triggered when the force reached the triggering level. Figure 5B shows the mean MEP amplitude evoked by the TS alone for SIHI (Fig. 5B, left) and LIHI (Fig. 5B, right), respectively. Gray lines indicate individual data. Two‐way ANOVA for the MEP amplitude evoked by the TS alone revealed no significant effects of ISI and TRIGGER TIMING (ISI: *F*_1,5_ = 0.974, *P* = 0.347; TRIGGER TIMING: *F*_3,15_ = 0.384, *P* = 0.765). Additionally, there was no significant ISI × TRIGGER TIMING interaction (*F*_3,15_ = 0.685, *P* = 0.568).

**Figure 6.**
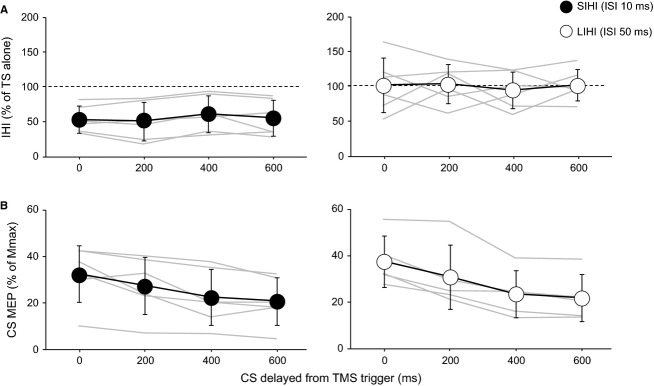
(A) Time courses of the mean SIHI and LIHI values (*n* = 6, ±SD) observed in the different timing of TMS trigger. Individual data from six subjects are shown in gray lines. The *y*‐axis indicates the conditioned MEP amplitude expressed as a percentage of the mean test MEP amplitude evoked by the TS alone showing the degree of IHI. The dashed lines indicate the MEP amplitude evoked by the TS alone (100%). Values below 100% indicate inhibition and values above 100% indicate facilitation. The *x*‐axis indicates the different timing of TMS trigger. (B) Time courses of the mean MEP amplitude values (SIHI:* n* = 6, ± SD; LIHI:* n* = 5, ±SD) evoked by the CS (CS MEP) observed in the different timing of TMS trigger. Individual data from six subjects for SIHI and five subjects for LIHI are shown in gray lines. Data from one subject for LIHI are not shown because constant visible CS MEPs were not obtained due to a low CS intensity for evoking LIHI. The *y*‐axis indicates the amplitude of CS MEP expressed as a percentage of maximum muscle response (Mmax). The *x*‐axis indicates the different timing of TMS trigger.

Figure 6A shows the mean IHI values recorded in the resting right FDI for both ISIs during the FM task. Two‐way ANOVA revealed a significant effect of ISI but no significant effect of TRIGGER TIMING on the degree of IHI (ISI: *F*_1,5_ = 10.886, *P* = 0.008; TRIGGER TIMING: *F*_3,15_ = 0.023, *P* = 0.995). There was no significant ISI × TRIGGER TIMING interaction (*F*_3,15_ = 0.899, *P* = 0.453). Figure 6B shows the mean CS MEP amplitude recorded in the active left FDI for both ISIs during the FM task. Unlike the mean IHI values, no significant effect of ISI but a significant effect of TRIGGER TIMING was found (ISI: *F*_1,4_ = 0.042, *P* = 0.842; TRIGGER TIMING: *F*_3,12_ = 38.332, *P* < 0.001). Additionally, there was no significant ISI × TRIGGER TIMING interaction (*F*_3,12_ = 0.331, *P* = 0.803). It should be noted that data from one subject for LIHI are not shown because we were unable to obtain constant and visible CS MEPs due to a low CS intensity (83.8% of RMT). Therefore, the mean CS MEP amplitude was analyzed in the remained five subjects. The results of Experiment 4 may indicate that the timing of TMS trigger during the FM task does not affect the excitability of the transcallosal inputs and the MEP amplitude evoked by the TS alone recorded in the resting FDI contralateral to the task‐performing hand. On the other hand, ANOVA and the individual data show that the MEP amplitude recorded in an active hand muscle is closely related to the timing of TMS trigger, as reported by previous studies (Lemon et al. 1995; Morishita et al. 2012).

## Discussion

The main findings of the present study were as follows: (1) significantly increased SIHI and decreased LIHI from the active to the resting M1 were observed during the FM task; (2) the different modulation of SIHI and LIHI during the FM task is not due to the stimulus intensity but dependent on the task conditions; (3) the changes in the MEP amplitude evoked by the TS alone and the degree of IHI during the FM task were not related to the timing of TMS trigger.

### Increased ipsilateral M1 excitability during FM task

A number of studies have reported that performing a unimanual motor task increases MEP amplitude recorded in resting hand muscles contralateral to the task‐performing hand (Stedman et al. 1998; Tinazzi and Zanette 1998; Muellbacher et al. 2000; Stinear et al. 2001; Ziemann and Hallett 2001; Perez and Cohen 2008; Morishita et al. 2011, 2012). The increased MEP amplitude was observed without modulating subcortical excitability (Stedman et al. 1998; Tinazzi and Zanette 1998) and transcallosal inputs from the active hemisphere have been suggested to have responsibilities for this phenomenon (Stedman et al. 1998; Tinazzi and Zanette 1998; Kobayashi et al. 2003). The result of increased ipsilateral M1 excitability during the FM task was consistent with our previous studies (Morishita et al. 2011, 2012) and previous studies that carried out complex unimanual motor tasks (Tinazzi and Zanette 1998; Ziemann and Hallett 2001). In order to reveal related mechanisms of increased ipsilateral M1 excitability during the FM task as a complex unimanual task, SIHI and LIHI were tested in the present study.

### Different modulation of SIHI and LIHI during FM task

Several studies have investigated the effects of performing a simple unimanual motor task on SIHI from the active to the resting M1 and it was found that SIHI was moderately increased (Ferbert et al. 1992; Perez and Cohen 2008; Vercauteren et al. 2008; Hinder et al. 2010; Uehara et al. 2014). The present results of SIHI were consistent with these previous studies and our previous study (Morishita et al. 2012). In the present study, decreased LIHI from the active to the resting M1 was also observed during the FM task. Previous studies showed no apparent modulation of LIHI from the active to the resting M1 during a simple unimanual motor task (Talelli et al. 2008a; Uehara et al. 2014). However, a methodological issue should be discussed here because one previous study showed decreased LIHI from the active to the resting M1 during the performance of a simple unimanual motor task (Nelson et al. 2009). The main difference between the previous study and the present study is that they adjusted the CS intensity during the task to evoke similar MEP amplitude to that evoked during the resting state. These two different ways of measuring IHI – procedures with or without CS intensity adjustment – apparently lead to different results, therefore, the methodological issue cannot be ignored. Although this issue is still under the debate, several lines of evidence suggest that using procedures without CS intensity adjustment may be appropriate and reliable. In our previous study, we reported different degrees of SIHI during hand motor tasks even though similar CS MEP amplitude in the active muscle was obtained (Morishita et al. 2012). Chen et al. (2003) and Ni et al. (2009) also found no differences in the degree of SIHI and LIHI, respectively, even though they obtained different CS MEP amplitude with different current directions of the CS. It is well known that different current directions of the magnetic coil evoke different MEP amplitude in the contralateral hand muscles (Sakai et al. 1997). Therefore, the results of Chen et al. (2003) and Ni et al. (2009) suggest that simply relying on MEP amplitude in hand muscles to decide the CS intensity for measuring IHI may be problematic. Additionally, the results of Experiment 4 support this view; we did not find the effects of timing of TMS trigger on SIHI and LIHI during the FM task. Although the task speed depends on the subjects, which is a limitation of the present study, the results showed clear differences in CS MEP amplitude between the trigger timing at 0 and 600 msec as we can see in Figure 6B. Large MEP amplitude was obtained in the active left FDI when the CS was delivered at 0 msec, however, these results were unlikely related to the degree of SIHI and LIHI (Fig. 6A). Finally, the results of Experiment 3 further support the findings of the present study by excluding the CS intensity as a potential factor for the different modulation of SIHI and LIHI; the results were dependent on the task conditions but the CS intensity. These results and the findings of previous studies support the assertion that the transcallosal and the corticospinal projections are distinct (Catsman‐Berrevoets et al. 1980; Lee et al. 2007), and suggest that we should use the same CS intensity among conditions. We conclude that at least our procedures for measuring IHI in the present study were appropriate and reliable.

It should be kept in mind that performing the pFM task likely leads to modulation of SIHI and LIHI as well, although the changes are subtle compared with those observed during the FM task. It is possible that performing a more complex task such as the FM task could lead to larger modulation of SIHI and LIHI. Unimanual motor tasks result in activation in both contralateral and ipsilateral hemispheres and complex unimanual motor tasks lead to larger activation in several motor‐related regions compared with simple unimanual motor tasks (Hummel et al. 2003; Tsuda et al. 2009). Thus, it is possible that the property of the FM task – the fine finger control and sensitive touch demanded by the FM task – led to larger activation in motor‐related regions. The pFM task does not demand the fine finger control and sensitive touch, it only requires a certain amount of force production. Therefore, it can be assumed to induce lower activation in motor‐related regions. Another important aspect that should be considered is that the FM task involves tool manipulation. Using tools is considered as complex because not only high levels of dexterity but also higher cognitive functions are required (Lewis 2006; Frey 2008). It was also reported that larger activation in several motor‐related regions were seen during tool manipulation (Tsuda et al. 2009), which was a similar task to the FM task in the present study. We suggest that the modulation of SIHI and LIHI from the active to the resting M1 observed in the present study may not only be due to voluntary activity but at least partially to larger activation in motor‐related regions derived from complexity of the task – task‐dependent modulation.

### Possible mechanisms of task‐dependent modulation of IHI

The mechanisms of task‐dependent modulation of IHI are unclear, however, several possible mechanisms can be thought. One possibility is that the modulation of SIHI and LIHI from the active to the resting M1 influences excitability of the M1 ipsilateral to the task‐performing hand. Daskalakis et al. (2002) reported that SIHI inhibits short‐interval intracortical inhibition in the target M1. Therefore, increased SIHI from the active to the resting M1 could be responsible for inhibiting short‐interval intracortical inhibition in the ipsilateral M1 (Morishita et al. 2012). As a result, decreased short‐interval intracortical inhibition in the ipsilateral M1 leads to increased ipsilateral M1 excitability, which was observed in Experiment 1. In fact, we reported decreased short‐interval intracortical inhibition in the ipsilateral M1 during the FM task compared with that observed during the pFM task in our previous study (Morishita et al. 2011). Udupa et al. (2010) reported that LIHI did not affect the degree of short‐interval intracortical inhibition in the target M1. Thus, it is possible that decreased LIHI from the active to the resting M1 directly influences ipsilateral M1 excitability. Another possible mechanism is related to the role of IHI as discussed in previous studies. It is believed that SIHI might play a crucial role in suppressing involuntary activity (Hübers et al. 2008). In accordance with this view, SIHI from the active to the resting M1 has to increase in order to suppress involuntary contralateral activity (Kobayashi et al. 2003) that can be seen more often during the performance of a demanding task (Hoy et al. 2004). Although involuntary contralateral activity during the performance of a complex unimanual task has not been systematically examined, we occasionally observed involuntary EMG activity in the resting right FDI during the FM task, which was excluded from the analysis in the present study (please see Experimental Procedure section). On the other hand, the role of LIHI has not been reported. Bologna et al. (2012) showed relationships between a reduction in induced mirror activity by training and the degree of SIHI. However, the degree of LIHI was unlikely related to any parameters regarding induced mirror activity.

From a different point of view, a relatively long latency of LIHI suggests that its modulation might reflect an involvement of other motor‐related regions. It was demonstrated that posterior parts of the corpus callosum were closely related to the degree of SIHI (Wahl et al. 2007; Li et al. 2013). Li et al. (2013) showed that patients with callosal infarction especially posterior parts of the corpus callosum exhibited less SIHI. Interestingly, although less LIHI was shown in patients with callosal infarction as well, any specific parts of the corpus callosum were unlikely related to the degree of LIHI. Thus their results could suggest that LIHI involves other responsible regions aside from the corpus callosum. Consistent with this evidence, Talelli et al. (2008a) showed a correlation between advancing age and modulation of LIHI during a simple unimanual motor task. They suggested that the results were probably related to activity in broad areas seen more often in older individuals (Ward 2006), meaning less LIHI in older individuals could potentially suggest an involvement of other motor‐related regions (Talelli et al. 2008a). In fact, a correlation between task‐related activity of supplementary motor area and modulation of LIHI during a simple unimanual motor task was found (Talelli et al. 2008b). Thus, it is possible that induced larger activation in motor‐related regions led to the modulation of LIHI during the FM task. Further investigation is required to confirm the explanations mentioned earlier.

## Conclusions

The present study examined the effects of performing a complex unimanual motor task on SIHI and LIHI. The results demonstrated the different modulation of SIHI and LIHI from the active to the resting M1 during the performance of the complex unimanual task. Only a few studies examined changes in LIHI from the active to the resting M1 during a unimanual motor task, and the present study is the first to demonstrate different modulation of SIHI and LIHI from the active to the resting M1 during a complex unimanual motor task. The results of the present study may suggest that SIHI and LIHI are implemented in distinct circuits with different functional meaning. The results might also suggest a relevant function of LIHI for effective motor control especially during a complex hand motor task. Functional relevance of modulation of SIHI and LIHI, such as relationships between electrophysiological and behavioral measures, will be investigated in the future studies.

## Conflict of Interest

None declared.
